# Shear-Wave Elastography for the Diagnosis of Pediatric Hashimoto’s Thyroiditis: A Systematic Review and Meta-Analysis

**DOI:** 10.7759/cureus.35490

**Published:** 2023-02-26

**Authors:** Trevor Decker, Emma Schnittka, Laurence Stolzenberg, Joel Yalowitz

**Affiliations:** 1 Radiology, Alabama College of Osteopathic Medicine, Dothan, USA; 2 Medicine, Alabama College of Osteopathic Medicine, Dothan, USA; 3 Orthopaedic Surgery, Alabama College of Osteopathic Medicine, Dothan, USA; 4 Radiology, Decatur Morgan Hospital, Decatur, USA

**Keywords:** diagnostic elastography, elastography, shear-wave ultrasonography, shear-wave elastography, pediatrics, hashimoto’s thyroiditis, diagnostic radiology, autoimmune thyroiditis

## Abstract

Shear-wave elastography (SWE) has emerged as a novel ultrasonographic technique for the diagnosis of pediatric Hashimoto’s Thyroiditis (HT). This systematic review and meta-analysis aim to summarize current evidence to determine the diagnostic value of SWE for HT. MEDLINE, a comprehensive search yielded five studies inclusive of 392 subjects. A meta-analysis comparing SWE values (kPa) between children with HT and healthy controls yielded a Cohen’s d-value of 1.34 (CI 1.02-1.65), suggesting statistically significant differences in SWE values. Such evidence indicates that SWE may serve as a valuable tool in diagnosing HT in the pediatric population.

## Introduction and background

Hashimoto’s thyroiditis (HT), also known as autoimmune thyroiditis, is a disease characterized by decreased thyroid function secondary to self-generated antibodies against the thyroid gland. The disease begins with lymphocytic infiltration and eventually progresses to irreversible fibrosis and subsequent loss of thyroid functionality. HT is the most common cause of hypothyroidism in the United States [1]. Additionally, HT is the most common thyroid disorder among children, affecting approximately 1-2% of the pediatric population [2, 3]. Early and accurate diagnosis of thyroid disorders is essential in this population to ensure proper physical and cognitive development [4].

Currently, the diagnosis of HT in children is based largely on clinical findings. Myxedema (swelling secondary to glycosaminoglycan deposition), dry hair and skin, fatigue, weight gain, and cold intolerance can all be present. Additionally, HT may cause growth retardation and bone age retardation in prepubescent children. However, these symptoms are nonspecific and further laboratory testing is needed to make a definitive diagnosis. Laboratory findings include elevated thyroid stimulating hormone (TSH) levels, low levels of free thyroxine (T4), and/or the presence of autoantibodies against thyroid peroxidase (anti-TPO) or thyroglobulin (anti-TG) [1]. While an elevated TSH is considered diagnostic of HT, it is important to note that this marker can be elevated secondary to other conditions, including obesity, infection, and excess iodine intake [5, 6]. Additionally, there have been reports of patients with clinically evident disease but negative serology [1]. Furthermore, 10-15% of the general population is positive for anti-TPO antibodies without any clinical symptoms [3]. Because of such ambiguity, further evaluation of the thyroid via grey-scale ultrasound is often utilized. Historically, ultrasonography has revealed areas of hypoechoic echogenicity and heterogeneous echotexture for this pathology. Yet, this “moth-eaten” appearance is only present in approximately one-third of children with HT and may not appear until later in the disease course [1, 7].

To overcome the lack of specificity in current diagnostic protocols, a novel ultrasound method, shear-wave elastography (SWE), has been explored as a potential tool for the evaluation of HT. SWE uses an acoustic radiofrequency impulse to vibrate a region of interest (ROI) within the targeted tissue. A measurement of pressure is then recorded in real-time to determine the elasticity of the tissue (given in kPa). A higher pressure or intensity of vibration correlates with a higher SWE value and reflects a loss of elasticity and/or the development of fibrosis. SWE has been found to have high intra- and interobserver correlation with good reproducibility and is thus considered “user-independent” [8]. The homogeneity of fibrosis within an individual thyroid gland is still debated by experts, however, the intraobserver variability of SWE values in Hashimoto's patients was found to range from 1.1% to 1.14% [8-10]. While current research recognizes the benefit of SWE in diagnosing adult patients with HT, only a few studies have explored the role of SWE in diagnosing HT in children [10, 11]. Through this systematic review and meta-analysis, we aim to synthesize data from available studies to determine if SWE may play a valuable role in the diagnosis of pediatric HT.

## Review

Materials and methods

Literature Search

**Figure 1:**
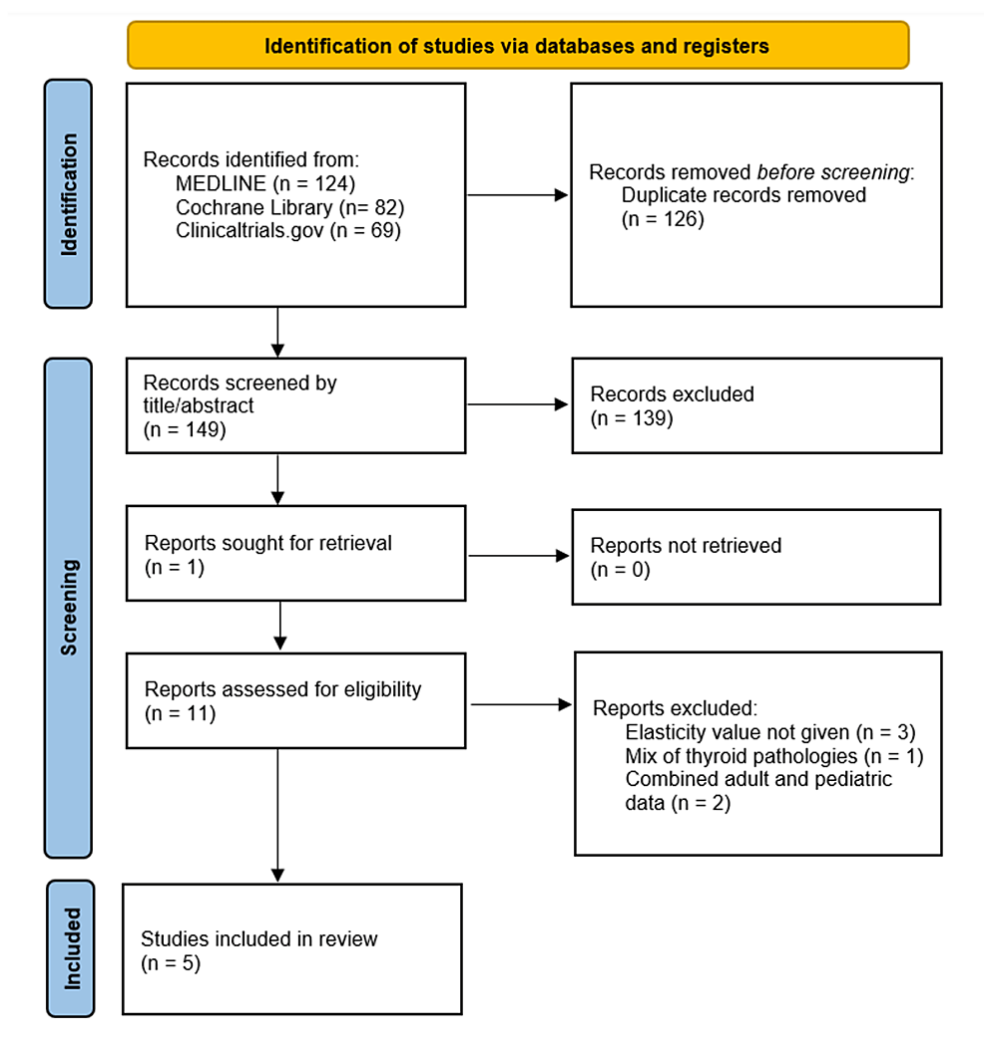
PRISMA 2020 Flow Diagram for Systematic Reviews

The Preferred Reporting Items for Systematic Reviews and Meta-Analyses (PRISMA) 2020 checklist guided our review [12]. MEDLINE, Cochrane Library, and Clinicaltrials.gov were searched. Databases were searched by two authors (TD and ES) from inception through September of 2022. First MEDLINE was accessed via PubMed. Both medical subject headings (MeSH) and free-text searches were utilized. Search terminology included the following: (("Hashimoto Disease"[MeSH]) AND ("Elasticity Imaging Techniques"[MAJR]) AND "Child"[MeSH])), (shear wave elastography) AND (Hashimoto) OR (autoimmune), and (shear wave elastography) AND (thyroid) AND (pediatric). Cochrane Library via Wiley was then searched using free-text searches with (shear wave elastography) +/- (Hashimoto) OR (autoimmune). Finally, a search of Clinicaltrials.gov was conducted using “shear wave elastography” and Hashimoto thyroiditis or autoimmune thyroiditis as designated conditions or diseases. Searches were not limited by date of publication or study type (Ex., Randomized controlled trials, meta-analyses, etc.). A flowchart of our literature search is detailed in Figure 1.

Quality Assessment and Data Extraction

Search results were then filtered by title and abstract resulting in the selection of 11 articles for full-text review. Studies were evaluated by two authors (TD and ES) independently for inclusion and exclusion criteria. Included studies were (1) limited to a pediatric population (defined as ages < 18), (2) utilized shear wave elastography as the primary diagnostic tool, (3) evaluated patients with Hashimoto’s thyroiditis, (4) provided elasticity data in the form of kilopascals (kPa), and (5) were available in English. Excluded studies (1) combined adult and pediatric data or only evaluated adults (defined as >18 years of age), (2) used alternative ultrasonography diagnostic methods (Ex., Strain wave elastography), (3) incorporated non-Hashimoto's thyroid pathology, and (4) analyzed parameters other than elasticity (Ex., Shear wave velocity).

Articles complying with selection criteria were then assessed for quality using the Joanna Briggs Institute checklist [13]. Reviewers (TD and ES) independently completed this checklist which offers 11 question prompts regarding publication quality. Individual assessments were then compared for comprehensive analysis. Next, reviewers extracted key information from each study. Key information for our review included authors, year of publication, number of participants in experimental and control groups, definitions for failure in void trials, and experimental findings. This data was extracted from included studies and compiled into a shared database for comparison.

Meta-Analysis

Meta-analysis calculations were made using Meta Essentials Excel Workbook [14]. We selected Workbook 3: Differences between independent groups-continuous data based on the format of raw data presented in included studies. Study heterogeneity and publication bias were quantitatively evaluated via p-value, I-value, Egger Regression Test, and Begg and Mazumdar Rank Correlation Tests.

Results

Study Characteristics

Five studies complied with all inclusion and exclusion criteria and were inclusive of 392 subjects - 223 in the experimental group and 169 in the control group. These studies spanned four countries and were published from 2018 to 2022. All included studies evaluated pediatric participants using similar diagnostic protocols with “healthy” controls defined as those negative for clinical signs and laboratory markers for HT. Participants in the “Hashimoto’s group” were positive for a combination of ultrasound findings, elevated TSH levels, the presence of anti-TPO or anti-TG antibodies and/or clinical signs of hypothyroidism.

All studies followed a prospective case-control format apart from Kandemirli et al. (retrospective case-control study) [15]. All studies found significantly higher SWE values in patients with HT. Additional thyroid parameters were also measured. Three studies found significantly higher thyroid volume in HT [15-17], two higher TSH levels [7, 8], four higher anti-TPO levels [7, 8, 16, 17], and three anti-TG levels [7, 16, 17]. Additional study characteristics are detailed in Table 1.

**Table 1:** Literature Summary EG: Experimental Group, CG: Control Group, SD: Standard Deviation, SWE: Shear-Wave Elastography Calculation, TSH: Thyroid-Stimulating Hormone, anti-TPO: Anti-Thyroid Peroxidase Antibodies, anti-TG: Anti-Thyroglobulin Antibodies, YE: Years’ Experience

Author (Year)	Country	Study Type	Radiology	Notes
Agarwal et al. (2021) [8]	India	Prospective case-control	Performer(s): 1 pediatric radiologist, 10 YE; Machine(s): Aixplorer Multiwave; Method: 4-15 MHz, 1 mm measurements, average of 3 measurements	Age range 4-12 years. Significantly higher TSH (P < 0.001), anti-TPO (P < 0.001), and SWE (P < 0.001) in EG. Noted some patients with HT had begun treatment (% unspecified).
Cepeha et al. (2021) [16]	Switzerland	Prospective case-control	Performer(s): not specified; Machine(s): Aixplorer Mach 30; Method: 4-18 MHz, measurement size not specified, average of 6 measurements	Age range 5-18. Significantly higher thyroid volume (p = 0.004), anti-TPO (P < 0.001), anti-TG (p = 0.001), and SWE (P < 0.001) in EG, 34% of EG undergoing thyroid replacement therapy. Compared SWE for children and adults with HD, significantly lower values in children (P < 0.001).
Hazem et al. (2021) [7]	Egypt	Prospective case-control	Performer(s): 1 radiologist, 17 YE; Machine(s): Xario 200 Platinum; Method: 3-11 MHz, 8-20 mm measurements, 5 measurements per lobe, average of 10 measurements	Age range not specified. Claim SWE values can differentiate between types of thyroid disease, P < 0.05. Significantly higher SWE (P < 0.05), TSH (P = 0.025), anti-TG (P = 0.006) and anti-TPO (P = 0.035) in EG. 78% of patients had previously received therapy.
Kandemirli et al. (2018) [15]	Turkey	Retrospective case-control	Performer(s): 2 pediatric radiologists, 10 YE and 2 YE; Machine(s): Aplio 500; Method: 14 MHz setting, 3 mm measurements, average of 6 measurements	Age range not specified. Significantly higher SWE (P < 0.001), thyroid volume (P < 0.001) in EG. Positive correlation between SWE value and anti-TPO levels (P < 0.05) in EG. Significantly higher SWE with disease progression (comparative of % thyroid involvement, p < 0.05).
Koca and Seber (2022) [17]	Turkey	Prospective case-control	Performer(s): 1 pediatric radiology, 12 YE; Machine(s): Aplio 500; Method: 14 MHz, 2 mm measurements, average of 10 measurements	Age range 5-17. Significantly higher SWE, thyroid volume, anti-TPO, anti-TG and thyroglobulin in EG (all P < 0.001). Identified positive correlation between BMI and SWE score in CG only (p = 0.02). EG diagnosed and untreated HT patients.

Analysis of Bias

Further assessment of bias was made through Egger Regression and Begg and Mazumdar Rank Correlation Tests which revealed p-values of 0.172 and 0.142, respectively. With both values greater than 0.05, we can assume minimal publication bias.

Meta-Analysis

A meta-analysis inclusive of SWE values with standard deviations from experimental and control groups yielded a Cohen’s d-value of 1.34 (CI 1.02-1.65). A value exceeding 1.0 indicates a statistically significant increase in SWE values for the diseased population. Furthermore, a confidence interval range which does not intersect 1.0 refutes the null hypothesis. Z-value of 11.74 was also calculated, further indicating a significant difference in elasticity between groups. Data used for meta-analysis calculations is displayed in Table 2.

**Table 2:** Meta-Analysis Data EG: Experimental Group, CG: Control Group

Author (Year)	EG (n1)	EG Elasticity (kPa +/- SD)	CG (n2)	CG Elasticity (kPa +/- SD)	P-Value
Agarwal et al. (2021) [8]	18	23.77 +/- 8.99	27	13.13 +/- 4.76	< 0.001
Cepeha et al. (2021) [16]	50	15.51 +/- 4.76	50	10.41 +/- 2.01	< 0.0001
Hazem et al. (2021) [7]	50	15.31 +/- 2.95	20	10.9 +/- 1.78	Not given (<0.001)
Kandemirli et al. (2018) [15]	59	14.9 +/- 3.63	26	10.6 +/- 1.7	<0.001
Koca and Seber (2022) [17]	46	12.94 +/- 6.01	46	8.23 +/- 2.82	<0.001
Combined Values	223	16.47	169	10.65	

Discussion

The results of this systematic review and meta-analysis reveal a significant difference in SWE values for children with HT compared to healthy controls. Such results verify the conclusions of previous research demonstrating SWE’s value in the diagnosis of HT and relate these findings to the pediatric population [11, 12]. We believe this review to be inclusive of all available data and the subsequent results to be valid and applicable to modern clinical practice.

Yet our review process was not without limitation. Perhaps the most confining factor was the lack of available data. Though our initial search yielded 149 articles for title and abstract screening, filtration resulted in just 11 articles eligible for full review. Despite this small pool, significant homogeneity was recognized across included studies. Each study incorporated a similar, comprehensive assessment for the diagnosis of HT, including combinations of laboratory markers, grey-scale ultrasonic findings, and clinical symptoms. Further continuity was recognized in SWE application and analysis. All studies analyzed thyroid elasticity using multiple ROI measurements. Results were presented with a mean SWE value (in kPa) and standard deviation (SD). The exception to this formatting was the study by Kandemirli et al. which listed interquartile range (IQR) rather than SD [15]. IQR values were converted to SD to limit heterogeneity, which was achieved, as depicted by a p-value of 0.419, though we acknowledge the possible inaccuracy of using converted data.

Additional limitations include possible researcher bias and the intrinsic limitations of SWE application. Notably, none of the included studies were blinded, with both researchers and technicians cognizant of which children were assigned to experimental and control groups. This lack of anonymity carries significant potential for bias. Furthermore, we recognize the possible limitation of using SWE for the diagnosis of HT, as it is dependent on the degree of fibrosis in the thyroid gland. This diffuse, fibrotic reaction may take up to four years to occur; therefore, SWE may not be useful in detection of subclinical HT [18].

Our analysis had originally hoped to determine diagnostic cut-off values of SWE to further analyze the clinical value of this technique; unfortunately, only two studies reported this information. Cepeha et al. found a cut-off value of 12.2 kPa yielded a sensitivity of 82% and specificity of 88% for diagnosing chronic autoimmune thyroiditis in children [16]. Similarly, Kandemirli et al. noted a cut-off value of 12.3 kPa afforded a sensitivity of 86.4 and specificity of 96.3% [15]. Combined data from the studies included in this analysis found average SWE values of 16.47 and 10.65 in the experimental and control groups, respectively, which correlates with these cut-off values.

## Conclusions

Despite these limitations, we believe the results of our analysis to bear weight within the clinical setting. Included publications were thoroughly assessed for quality and meta-analysis calculations reveal significant differences in SWE values for children with and without HT. Ultrasonography has long been appreciated for its usability, real-time capability, and low cost and we believe advances in this technology through the development of SWE to serve as a tremendous asset within diagnostic radiology. This review serves as evidence supporting the use of SWE in diagnosing children with HT. We encourage further research on this topic to validate our findings and to ensure the proper diagnostic protocols for thyroid pathology within the pediatric population.
